# Latent Class Analysis of Negative Emotion Subtypes and Their Association With Quality of Life in Patients With Pituitary Neuroendocrine Tumors

**DOI:** 10.1002/brb3.71644

**Published:** 2026-08-02

**Authors:** Lei Jin, FangYuan Yao, JinHua Yang, Jing Gong, JingHua Sheng, ShuYa Chen, Li Ji, Chen Wei

**Affiliations:** ^1^ Department of Nursing Huashan Hospital of Fudan University Shanghai China

**Keywords:** latent class analysis, negative emotions, pituitary neuroendocrine tumors, quality of life

## Abstract

**Introduction:**

Pituitary neuroendocrine tumors (PitNETs) account for up to 15% of intracranial tumors. Patients commonly present with various emotional and cognitive impairments, including a high prevalence of anxiety and depression. Therefore, this study aimed to identify the latent subtypes of negative emotions in patients with PitNETs and examine their associations with quality of life.

**Methods:**

A total of 422 patients diagnosed with PitNETs who were scheduled for neurosurgical treatment at a tertiary hospital between September 2023 and September 2025 were enrolled. Multidimensional assessments were conducted, including a general information questionnaire, the Hospital Anxiety and Depression Scale (HADS), the 12‐Item Short Form Health Survey (SF‐12), the Pittsburgh Sleep Quality Index (PSQI), and neuroendocrine hormonal function classification. Latent class analysis was applied to identify subtypes of negative emotions, and multivariable regression was performed to compare psychological and clinical characteristics across classes.

**Results:**

Three distinct latent subtypes of negative emotions were identified: the low negative‐emotion class (C1; *n* = 75, 18%); moderate‐distress class (C2; *n* = 218, 52%); and high dual‐negative‐emotion class (C3; *n* = 129, 30%). Multivariable regression showed significant differences between C1 and C3 in educational level, employment status, alcohol consumption, headache symptoms, and PSQI scores (*p* < 0.05). Significant differences were also found between C2 and C3 in pituitary hormone function, headache symptoms, and PSQI scores (*p* < 0.05).

**Conclusion:**

Negative emotions in patients with PitNETs demonstrated marked heterogeneity. Therefore, identifying the distinct emotional subtypes may facilitate more precise psychological interventions, thereby improving quality of life and perioperative management.

## Graphical abstract

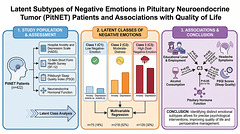

## 1 Introduction

Pituitary neuroendocrine tumors (PitNETs) originate from the neuroendocrine cells of the anterior pituitary gland and account for 8%–15% of intracranial tumors (Li et al. 2024). Patients commonly present with endocrine dysfunction, tumor mass‐effect symptoms, and varying degrees of emotional and cognitive impairments (Alexander et al. 2022). Emotional symptoms are of considerable significance in patients with PitNETs. The prevalence of negative emotions such as anxiety and depression among such patients is 50.00% and 31.54%, respectively, which is significantly higher than that of the general population (Pertichetti et al. 2020). Negative emotions not only affect the patient's psychological well‐being but may also interfere with treatment adherence, postoperative recovery, and overall quality of life. Therefore, psychological assessment and intervention warrant increased attention in clinical nursing practice.

Unlike those of primary psychiatric disorders, PitNETs‐related negative emotions stem from a series of complex, intertwined mechanisms. The tumor‐related disruption of the hypothalamic‐pituitary‐adrenal (HPA) axis can derange mood‐regulating hormones like cortisol, growth hormone, and prolactin (Chieffo et al. 2023; Pertichetti et al. 2020). In addition, mass effect in the sellar region may cause compression or distortion of adjacent limbic networks, including the hippocampus and amygdala, triggering various psychiatric manifestations (Burke et al. 2024; Gökoğlu et al. 2025). Because these somatic and hormonal alterations vary drastically across patients, the resulting presentation is highly heterogeneous. However, most studies on the psychological status of patients with PitNETs rely on mean comparisons or risk factor analyses, resulting in an insufficient focus on the heterogeneity of emotional characteristics. Thus, neglecting this internal variability may result in an incomplete understanding of the patients’ psychological needs.

Latent class analysis (LCA) is a person‐centered statistical approach that identifies unobserved subgroups based on individual response patterns in psychological assessments. This method provides an objective basis for revealing the distributional characteristics of negative emotions. By clarifying the features of different emotional subtypes, LCA may help researchers further explore the relationship between various emotional states and quality of life, offering valuable insights for nursing practitioners. Therefore, this study aimed to identify the latent subtypes of negative emotions in patients with PitNETs and examine their associations with quality of life to identify and address the patients’ psychological concerns.

## 2 Materials and Methods

### 2.1 Participants

Patients diagnosed with PitNETs who were scheduled to undergo transsphenoidal pituitary tumor resection at our institution between September 2023 and September 2025 were consecutively recruited. The inclusion criteria were: (1) diagnosis of PitNET with a planned surgical intervention; (2) consciousness and ability to communicate normally; and (3) willingness to participate and provide written informed consent.

The exclusion criteria were: (1) presence of malignancies in other organs; (2) current use of psychotropic medications that may affect emotional status; (3) history of craniotomy that may influence mental state; and (4) previously diagnosed psychiatric disorders.

This study was approved by the Huashan Institutional Review Board (HIRB, No.2023‐938).

### 2.2 Data Collection

#### 2.2.1 General Information

General information included demographic and clinical data. Demographic variables comprised age, sex, body mass index (BMI), smoking history, and alcohol consumption. Clinical information included history of chronic diseases and pituitary hormonal functional classification. To properly capture the somatic drivers of emotional symptoms, pain and sleep profiles were evaluated. Headache pain severity was quantified using the Pain Numerical Rating Scale (PNRS), an 11‐point scale (0 = no pain, 10 = worst imaginable pain) widely validated for rapid, reliable pain assessment in neurosurgical settings. Sleep quality was measured using the Pittsburgh Sleep Quality Index (PSQI), which evaluates seven sleep domains over a 1‐month lookback period. The PSQI is highly sensitive in identifying sleep‐wake cycle disruptions secondary to suprachiasmatic nucleus compression or neuroendocrine shifts in pituitary tumor cohorts (Carpi 2025).

#### 2.2.2 Measures

The Hospital Anxiety and Depression Scale (HADS) was used to assess anxiety and depression symptoms. The HADS consists of two subscales: the Hospital Anxiety Scale (HAS) and the Hospital Depression Scale (HDS) subscales (7 items each, scored 0–21 per subscale). The HADS is explicitly engineered for hospital medical wards, deliberately excluding confounding somatic indicators (such as fatigue or weight changes) to reflect true psychological distress in patients with organic diseases. A score of 8 or higher on either subscale was utilized as the cutoff point indicating clinically significant symptoms (Stern 2014; Zigmond and Snaith 1983).

Health‐related quality of life was assessed using the 12‐item Short‐Form Health Survey (SF‐12), which includes two domains: the Physical Component Scale (PCS) and the Mental Component Scale (MCS). Scores range from 0 to 100, with higher scores representing a better quality of life (Ware et al. 1996).

#### 2.2.3 Data Collection

Demographic and clinical information were extracted from electronic medical record. Data from the PNRS, PSQI, and HADS were gathered using standardized questionnaires. Consecutive patients admitted for transsphenoidal surgery were screened against eligibility criteria by two dedicated clinical research nurses before any preoperative interventions. Eligible participants received full study briefings and unified instructions for questionnaires before completing them in a questionnaires in a quiet, standardized ward setting. For patients with reading and visual difficulties, items were verbally read out by research nurses. All data collected were cross‐verified by two researchers.

### 2.3 Statistical Analysis

Statistical analyses were performed using SPSS version 27.0. Categorical variables were presented as frequencies and percentages, and group differences were assessed using the chi‐square test. Continuous variables were expressed as mean ± standard deviation (X̄ ± s), and group comparisons were conducted using the *t*‐test. Variables with statistically significant differences in univariate analyses were employed in a multivariable logistic regression model.

LCA of the HADS data was conducted using Mplus version 8.3. Model parameters were estimated using the maximum likelihood method, and individuals were assigned to latent classes based on the highest posterior probability. Model fit was evaluated using the Bayesian Information Criterion (BIC), adjusted BIC, and Akaike Information Criterion. Classification quality was assessed using average posterior probabilities (> 0.70) and entropy (> 0.80). The optimal number of classes was determined using the bootstrap likelihood ratio test (BLRT) and the Lo–Mendell–Rubin adjusted likelihood ratio test (LMRT). The latent classes reflected the underlying heterogeneity of the negative emotional profiles among patients.

## 3 Results and Discussion

### 3.1 Results

#### 3.1.1 General Characteristics

A total of 524 questionnaires were distributed, and 422 valid questionnaires were collected, yielding an effective response rate of 80.5%. General characteristics of the 422 patients are summarized in Table 1.

**TABLE 1:** General characteristics of patients with PitNETs (*n* = 422).

Variable		Category	*N* (%)
Sex	Male	205	48.6
	Female	217	51.4
Age (years)	< 45	88	20.9
	45–59	234	55.5
	≥ 60	100	23.7
BMI (kg/m^2^)	≤ 23.9	184	43.6
	> 24	238	56.4
Marital status	Single	58	13.7
	Married	364	86.3
Educational level	Below college	187	44.3
	College and above	235	55.7
Employment status	Employed	227	53.8
	Unemployed/retired	195	46.2
Childbearing history	No	77	18.2
	Yes	345	81.8
History of surgery	No	336	79.6
	Yes	86	20.4
Smoking history	No	359	85.1
	Yes	63	14.9
Alcohol consumption	No	330	78.2
	Yes	92	21.8
Chronic disease	No	292	69.2
	Yes	130	30.8
Tumor size	< 3 cm	281	66.6
	≥ 3 cm	141	33.4
Pituitary hormonal function	Nonfunctioning PitNET	174	41.2
	Functioning PitNET	248	58.8

Abbreviations: BMI, body mass index; PitNET, pituitary neuroendocrine tumor.

#### 3.1.2 Correlation Between Negative Emotions and Quality of Life in Patients With PitNETs

Among the 422 patients included in the analysis, the mean HAS score was 10.75 ± 4.74, mean HDS score was 9.64 ± 4.65, and mean total HADS score was 20.39 ± 9.01. HAS ≥ 8 was identified in 327 patients, and HAD ≥ 8 in 294 patients. The prevalence of anxiety and depression was 77.5% (327/422) and 69.7% (294/422), respectively. The HAS and HDS scores were strongly positively correlated (*r* = 0.844, *p* < 0.01), and both were highly correlated with the total HADS score (*r* = 0.961 and 0.959, respectively, *p* < 0.01). The mean PCS and MCS scores of the SF‐12 were 35.63 ± 31.39 and 27.68 ± 34.73, respectively, and they were strongly positively correlated (*r* = 0.913, *p* < 0.01). Pearson correlation analysis showed that HAS, HDS, and total HADS scores were negatively correlated with both PCS and MCS scores (*r* = −0.304 and−0.349, *p* < 0.01) (Table 2).

**TABLE 2:** Correlation analysis between negative emotions and quality of life in patients with PitNETs.

Variable	HAS	HDS	HADS	PCS	MCS
HAS	1				
HDS	0.844**	1			
HADS	0.961**	0.959**	1		
PCS	−0.341**	−0.328**	−0.349**	1	
MCS	−0.304	−0.308**	−0.319**	0.913**	1

Abbreviations: HADS, Hospital Anxiety and Depression Scale; HAS, Hospital Anxiety Scale; HDS, Hospital Depression Scale; MCS, Mental Component Scale; PCS, Physical Component Scale; PitNET, pituitary neuroendocrine tumor.

**p < 0.01.

#### 3.1.3 Latent Class Analysis of Negative Emotions in Patients With PitNETs

The three‐class model demonstrated the best fit for identifying latent subtypes of negative emotions in patients with PitNETs. The entropy value was 0.811 (> 0.80), and both the LMR test and BLRT yielded significant results (*p *< 0.01), supporting the superiority of the three‐class solution. The class proportions were clinically interpretable, and the average posterior probabilities for all classes exceeded 95%, indicating good model reliability and high classification accuracy (Table 3). The optimal model classified patients into three latent classes: Class 1 (C1; *n* = 75, 18%), characterized as the low negative‐emotion class; Class 2 (C2; *n* = 218, 52%), characterized as the moderate‐distress class; and Class 3 (C3; *n* = 129, 30%), characterized as the high dual‐negative‐emotion class.

**TABLE 3:** Model fit indices for latent class models of negative emotions in patients with PitNET.

M	*k*	AIC	BIC	aBIC	entropy	*p*		Class size (%)
						LMR	BLRT	
1	4	5010.90	5027.08	5014.39	—	—	—	—
2	7	4782.67	4810.99	4788.77	0.704	< 0.01	< 0.01	0.38/0.62
3	10	4623.37	4663.82	4632.08	0.811	< 0.01	< 0.01	0.18/0.52/0.30
4	13	4524.08	4576.66	4535.41	0.862	0.039	0.041	0.17/0.46/0.34/0.03
5	16	4503.88	4568.60	4517.82	0.783	0.595	0.617	0.13/0.23/0.28/0.34/0.03

Abbreviations: aBIC, adjusted Bayesian Information Criterion; AIC, Akaike Information Criterion; BIC, Bayesian Information Criterion; BLRT, bootstrap likelihood ratio test; LMR, Lo–Mendell–Rubin adjusted likelihood ratio test.

#### 3.1.4 Comparison of Quality of Life Among Different Negative Emotion Classes in Patients With PitNETs

**FIGURE 1:**
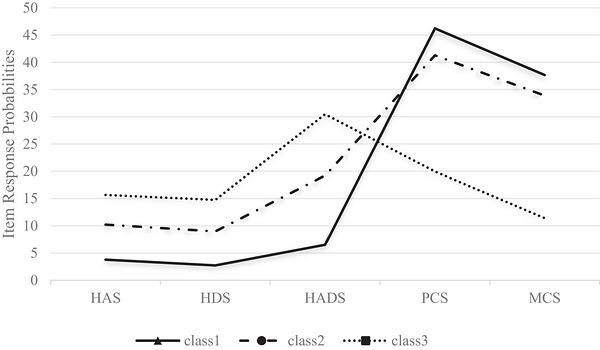
Item response probabilities for latent classes of negative emotions in patients with PitNETs. HADS, Hospital Anxiety and Depression Scale; HAS, Hospital Anxiety Scale; HDS, Hospital Depression Scale; MCS, Mental Component Scale; PCS, Physical Component Scale; PitNET, pituitary neuroendocrine tumor.

As summarized in Table 4, class C1 exhibited the lowest HAS, HDS, and total HADS scores (3.79 ± 2.54, 2.73 ± 2.02, and 6.52 ± 3.67, respectively) and the highest PCS and MCS scores (46.21 ± 25.88 and 37.66 ± 30.13, respectively). In contrast, Class C3 displayed the highest levels of negative emotions, with the highest HAS, HDS, and HADS scores (15.67 ± 2.87, 14.77 ± 2.65, and 30.44 ± 4.70, respectively), and the lowest PCS and MCS scores (19.95 ± 30.99 and 11.41 ± 33.47, respectively). The SF‐12 PCS and MCS scores in Class C3 were significantly lower than those in Classes C2 and C1 (*p *< 0.01). Class C2 demonstrated intermediate HAS, HDS, and HADS scores (10.23 ± 2.24, 8.98 ± 1.97, and 19.21 ± 3.46, respectively), with corresponding PCS and MCS scores also falling between those of C1 and C3 (41.26 ± 30.00 and 33.88 ± 33.75, respectively), showing significantly lower scores compared with Class C1 (*p *< 0.01) (Figure 1).

**TABLE 4:** Comparison of quality of life among different negative emotion classes in patients with PitNETs.

Variable	HAS	HDS	HADS	PCS	MCS
Low negative‐emotion class (C1)	3.79 ± 2.54	2.73 ± 2.02	6.52 ± 3.67	46.21 ± 25.88	37.66 ± 30.13
Moderate‐distress class (C2)	10.23 ± 2.24	8.98 ± 1.97	19.21 ± 3.46	41.26 ± 30.00	33.88 ± 33.75
High dual‐negative‐emotion class (C3)	15.67 ± 2.87	14.77 ± 2.65	30.44 ± 4.70	19.95 ± 30.99	11.41 ± 33.47
*F*	547.02	723.416	905.090	26.786	22.88
*p*	< 0.01	< 0.01	< 0.01	< 0.01	< 0.01

Abbreviations: HADS, Hospital Anxiety and Depression Scale; HAS, Hospital Anxiety Scale; HDS, Hospital Depression Scale; MCS, Mental Component Scale; PCS, Physical Component Scale; PitNET, pituitary neuroendocrine tumor.

#### 3.1.5 Factors Associated With Latent Classes of Negative Emotions in Patients With PitNETs

Using the three latent classes of negative emotions as the dependent variable, univariate analysis was conducted on 14 independent variables. Six variables—employment status, educational level, alcohol consumption, pituitary hormonal function, headache, and PSQI score—showed significant associations (*p* < 0.05). These six variables were subsequently included in a multivariable logistic regression model. The three‐class latent variable was used as the dependent variable (with Class C3 as the reference category), and variables with *p* < 0.05 in the univariate analysis were used as independent variables. Variable coding is shown in Table 5. The results indicated that significant differences were observed between Class C1 and Class C3 (*p* < 0.05) in educational level (OR* = *2.631, *p = *0.039), employment status (OR* = *0.387, *p = *0.035), alcohol consumption (OR* = *3.118, *p = *0.026), headache symptoms (OR* = *2.595, *p *< 0.01), and PSQI scores (OR* = *0.910, *p *< 0.01). In addition, significant differences between Class C2 and Class C3 were found in pituitary hormonal function (OR* = *0.498, *p *< 0.012), headache symptoms, and PSQI scores (*p* < 0.05) (Table 6.).

**TABLE 5:** Variable coding for logistic regression analysis.

Independent variable	Coding
Educational level	0 = Below college; 1 = College and above
Employment status	0 = Unemployed/retired; 1 = Employed
Alcohol consumption	0 = No; 1 = Yes
Pituitary hormonal function	0 = Nonfunctioning PitNET; 1 = Functioning PitNET
Headache	0 = No; 1 = Yes
PSQI score	Entered as a continuous variable

Abbreviations: PitNET, pituitary neuroendocrine tumor; PSQI, Pittsburgh Sleep Quality Index.

**TABLE 6:** Factors associated with latent classes of negative emotions in patients with PitNETs.

Variable			Univariate analysis	Multivariate analysis			
				C1–C3		C2–C3	
			*p*	OR	*p*	OR	*p*
Sex	Male		0.241				
	Female						
Age (years)	< 45		0.400				
	45–59						
	≥ 60						
BMI (kg/m^2^)	< 18.5		0.431				
	18.5–24						
	> 24						
Marital status	Single		0.614				
	Married						
Educational level	Below college		0.034*	2.631	0.039*		
	College and above						
Employment status	Employed		0.015*	0.387	0.035*		
	Unemployed/retired						
Childbearing history	No		0.342				
	Yes						
History of surgery	No		0.358				
	Yes						
Smoking history	No		0.732				
	Yes						
Alcohol consumption	No		0.016*	3.118	0.026*		
	Yes						
Chronic disease	No		0.655				
	Yes						
Tumor size	< 3 cm		0.027*			0.498	0.012*
	≥ 3 cm						
Preoperative symptoms	Headache	No	0.002*	2.141	0.046*	2.595	< 0.01*
		Yes					
	Visual impairment	No	0.510				
		Yes					
	Menstrual irregularities	No	0.455				
		Yes					
	Changes in appearance	No	0.634				
		Yes					
PSQI score			< 0.01*	0.804	< 0.01*	0.910	0.003*

*Note*: For educational level, employment status, alcohol consumption, pituitary hormonal function, headache, and PSQI score, the reference category for categorical variables corresponds to those coded as 0.

Abbreviations: BMI, body mass index; PitNET, pituitary neuroendocrine tumor; PSQI, Pittsburgh Sleep Quality Index.

* *P*<0.05

### 3.2 Discussion

#### 3.2.1 Correlation Between Negative Emotions and Quality of Life in Patients With PitNETs

A high proportion of patients with various types of PitNETs experience negative emotions. For instance, the prevalence of anxiety in patients with prolactinomas has been reported to be 59% (Miao et al. 2024), while among those with growth hormone‐secreting adenomas (acromegaly), anxiety rates can reach up to 80% (Pertichetti et al. 2020). Patients with Cushing's disease exhibit depression rates ranging from 50% to 80% (Pertichetti et al. 2020), and the overall prevalence of psychological disturbances—including emotional instability and social anxiety—may be as high as 80% (Chieffo et al. 2023). Even in cases of nonfunctioning PitNETs, anxiety prevalence has been reported to range from 30% to 50% (Chieffo et al. 2023). Our study demonstrated a high overall baseline prevalence of anxiety (77.5%) and depression (69.7%), which sit at the upper end of historical reports. This result is likely attributable to the fact that our data were collected at a highly specialized, national‐level tertiary referral neurosurgical center. Patients admitted here often exhibit advanced tumor mass effects, severe endocrine dysfunction, or significant headache symptoms. Human emotions are primarily regulated by the limbic system, a core brain network encompassing the hippocampus, amygdala, and related limbic structures. Neuroimaging studies have demonstrated that volumetric changes in the lateral ventricle—which is anatomically adjacent to the limbic system—are closely related to affective disorders in various conditions, such as major depressive disorders, anxiety disorders, and so on. (Burke et al. 2024). Recent evidence has also shown significant atrophy of hippocampus and limbic regions in PitNET patients (Gökoğlu et al. 2025). This could be the result of mechanical compression from the mass effect in the sellar region or remodeling of brain parenchyma and the lateral ventricle due to cerebral spinal fluid (CSF) circulation disorders, such as hydrocephalus. These neuroanatomical changes may provide the structural and functional basis for the emotional disorders observed in this study.

In the present study, the HAS and HDS scores were strongly positively correlated, and both were highly correlated with the total HADS score. In addition, the total HADS score and its subscales were significantly negatively correlated with the SF‐12 PCS and MCS scores, indicating that higher levels of severe anxiety and depression are associated with a poorer quality of life. PCS and MCS scores were also strongly positively correlated, reflecting the close alignment between physical and psychological health.

These findings are consistent with previous reports Pertichetti 2020[8], further confirming the persistent and detrimental impact of negative emotions on quality of life in patients with PitNETs. However, relying solely on overall correlation analyses limits the ability to capture the internal differences and hierarchical structure of psychological characteristics within the patient population. Given this heterogeneity, applying LCA was necessary to identify potential subtypes within the cohort, thereby providing more targeted and evidence‐based guidance for clinical nursing practice.

#### 3.2.2 LCA of Negative Emotions in Patients With PitNETs

Building on the overall correlation findings, LCA was further applied to uncover the underlying heterogeneity within the patient population and to identify distinct psychological subtypes. The optimal model classified patients into three latent classes: C1, characterized as the low negative‐emotion class; C2, characterized as the moderate‐distress class; and C3, characterized as the high dual‐negative‐emotion class.

Clear distinctions were demonstrated among the three classes in terms of anxiety, depression, and quality‐of‐life indicators. This classification pattern highlights the hierarchical differences in psychological profiles and provides a solid foundation for developing tailored nursing interventions. The distinct separation of probability curves not only supports the scientific rigor and clinical interpretability of the LCA‐derived classes but also underscores the methodological advantages of LCA over traditional mean‐comparison approaches. By revealing latent internal differences within the population, LCA offers a new perspective and pathway for advancing precision nursing care.

#### 3.2.3 Comparison of Quality of Life Across Different Negative Emotion Classes in Patients With PitNETs

Significant differences were observed across the latent classes of patients with PitNETs in terms of anxiety, depression, and all dimensions of quality of life. Patients in Class C1 exhibited the lowest HAS, HDS, and total HADS scores and the highest PCS and MCS scores, indicating relatively stable emotional status and better overall quality of life. Class C2 demonstrated intermediate HAS, HDS, and HADS scores, with corresponding PCS and MCS scores also falling between those of C1 and C3. In contrast, Class C3 displayed the highest levels of negative emotions, with the highest HAS, HDS, and HADS scores and the lowest PCS and MCS scores, indicating markedly elevated anxiety and depression and the most impaired quality of life.

These findings suggest a clear gradient distribution in both psychological status and quality of life among patients with PitNETs, where higher the level of negative emotions, poorer the quality of life. The differences observed across latent classes further validate the rationality of the LCA‐derived groupings and provide clearer targets for tailored nursing interventions.

#### 3.2.4 Factors Influencing the Latent Classes of Negative Emotions in Patients With PitNETs

The latent class membership of negative emotions among patients with PitNETs was influenced not only by psychological assessment results but also by several demographic and clinical variables. Univariate analysis indicated that educational level, employment status, alcohol consumption, pituitary hormonal function, headache, and sleep quality score were significantly associated with class distribution.

Multivariable logistic regression analysis, using the high negative‐emotion class (C3) as the reference group, revealed that a lower educational level (below college) was a protective factor associated with low negative emotions. This may be explained by the fact that patients with higher educational levels generally possess stronger abilities to obtain disease‐related information and perceive disease risks, leading them to experience greater uncertainty regarding a much longer perceived medical course. The “fear of the unknown” thereby might render them more prone to anxiety and depressive symptoms. Conversely, patients with lower educational attainment may rely more on medical decision‐making and pay less active attention to the complexity of the disease, thus presenting lower emotional burden in short‐term assessments.

Employment was associated with a reduced risk of high negative emotions; alcohol consumption increased the likelihood of higher negative emotions; and the absence of headaches significantly reduced the risk of high negative emotions. Sleep quality score was strongly associated with class distribution, suggesting that poorer sleep quality increased the probability of high negative emotions.

In addition, pituitary hormonal function significantly influenced the classification between the moderate‐distress (C2) and high negative‐emotion (C3) classes. Existing studies have demonstrated that elevated levels of PRL, GH, and ACTH can impair patients’ quality of life. Depression is also commonly observed in patients with excessive GH and ACTH secretion, a finding that has been confirmed and extensively discussed in previous research (Pertichetti et al. 2020).

#### 3.2.5 Implications for Latent Class Analysis‐Based Differentiated Nursing Care

In describing emotional symptoms, there's no strict distinction between the terms negative emotion and psychological distress. Nevertheless, negative emotions are usually considered milder in severity and shorter in duration relative to psychological distress in literature. The present study emphasizes the term “negative emotion” because patients with PitNETs, particularly those undergoing their first surgery, can achieve timely relief from negative emotions through comprehensive treatment approaches, including precision nursing interventions. Such early mitigation can prevent the progression toward more persistent and severe state of psychological distress, and ultimately improve patients’ quality of life (Feng et al. 2023). From this perspective, the clinical value of differentiated nursing care for at‐risk patients is further highlighted.

First, a precision nursing framework based on latent class characteristics can be established. The identification of distinct latent classes (C2 and C3) among PitNETs patients suggests that a “one‐size‐fits‐all” nursing approach is inadequate for addressing the emotional heterogeneity of these patients. For Class C2 patients, our findings advocate for a preventative nursing model. Interventions should focus on early emotional screening, standardized disease education, and perioperative expectation management. By providing systematic sleep guidance and routine follow‐up, clinicians can effectively prevent the transition from subclinical distress to clinical emotional deterioration. For Class C3 patients, a more intensive, multidisciplinary nursing pathway is warranted. For these patients, psychological counseling should not be a standalone intervention but must be integrated with clinical symptom management. We propose that symptom control (particularly for headache and sleep disturbances) and psychological intervention be conducted simultaneously rather than sequentially, thereby rapidly reducing the emotional burden and improving the postoperative quality of life.

Second, targeted management of risk factors should be adopted. While education is generally expected to increase awareness and reduce anxiety, an unexpected finding of this study is that lower educational level appears to serve as a protective factor against high negative emotional states. This inverse correlation suggests that differentiated strategies should be implemented according to the cognitive characteristics of different educational groups. For patients with high education level, who typically possess higher information‐processing capabilities but are prone to uncertainty‐induced anxiety. Nursing staff should carefully guide them to build a rational cognitive framework by providing precise, data‐driven information, including detailed interpretations of diagnostic markers and surgical nuances, to alleviate anxiety derived from information gaps. On the other hand, for patients with lower education level, information transmission should be reasonably simplified. Key information can be reinforced in easily acceptable formats (oral notifications, graphic prompts) to help them establish reasonable expectations.

Our data also revealed a strong relationship between sleep and pain and latent class membership, indicating that prioritizing sleep and pain management may prove more effective than focusing solely on emotional regulation. For patients presenting with significant preoperative headache or sleep disorders, nursing assessments should be shifted forward early to symptom‐management level. Incorporating pain control, environmental optimization, and early multidisciplinary consultations into the routine pathway can be essential to reduce the risk of developing negative emotions. Furthermore, the impact of hormone levels on emotional states suggests that an early, comprehensive assessment covering both hormonal and emotional status is vital. Rational use of hormone‐adjusting drugs is needed to improve negative emotions in patients with high hormone levels.

In summary, educational level, employment status, alcohol consumption, headache, and sleep quality emerged as key factors affecting the latent classes of negative emotions in patients with PitNETs. Among these, sleep quality and alcohol consumption are modifiable lifestyle factors, while headache and endocrine dysfunction highlight the need for strengthened symptom management and hormonal regulation. These findings not only clarify the potential risk factors contributing to heightened negative emotions but also provide a foundation for developing targeted nursing approaches and offer actionable strategies for clinical nursing practice.

This study had some limitations. First, owing to the single‐center design implemented at Huashan Hospital in Shanghai, the findings may not be readily generalizable to PitNET patients from diverse cultural, social, and healthcare contexts. Second, the study employed a cross‐sectional design, which precludes a definitive causal relationship regarding the observed associations. Third, patients with a history of craniotomy were excluded, as prior neurosurgery, perioperative experiences, and risk perceptions of retreatment may exert complex effects on patients’ emotional state and coping styles, which would confound the validity of the latent variable analysis. Nevertheless, this exclusion may lead to selection bias. In addition, unmeasured factors may exist. Future multicenter longitudinal studies are needed to further validate and refine targeted nursing strategies.

## 4 Conclusion

This study found a significant negative correlation between negative emotions and quality of life in patients with PitNETs. Three distinct psychological subtypes were identified—low negative‐emotion class, moderate‐distress class, and high dual‐negative‐emotion class—indicating clear heterogeneity in the emotional profiles of these patients. Educational level, employment status, alcohol consumption, headache, and sleep quality were key factors influencing emotional class, among which sleep quality and alcohol use represent modifiable targets for intervention.

These findings reveal the hierarchical characteristics of psychological states in patients with PitNETs and provide empirical evidence for developing differentiated nursing treatment pathways. Applying such personalized approaches may help optimize nursing resource allocation, improve patients’ psychological well‐being and overall quality of life, and promote the transition from experience‐based to precision nursing.

## Author Contributions

**Lei Jin**: conceptualization, investigation, methodology, supervision, writing – review and editing. **FangYuan Yao**: data curation, formal analysis, investigation, software, validation, visualization, writing – original draft. **JinHua Yang**: data curation, investigation, software, validation, visualization. **Jing Gong**: data curation, investigation, software, validation. **JingHua Sheng**: data curation, investigation, software, validation. **ShuYa Chen**: data curation, investigation, software, validation. **Li Ji**: conceptualization, project administration, supervision, writing – review and editing. **Chen Wei**: conceptualization, funding acquisition, methodology, project administration, supervision, writing – review and editing.

## Funding

This work was supported by the Shanghai Municipal Commission of Health General Scientific Research Program (202340168).

## Ethics Statement

All procedures performed in this study involving human participants were in accordance with the ethical standards of the institutional and/or national research committee and with the 1964 Helsinki Declaration and its later amendments or comparable ethical standards. The study was approved by the Huashan Institutional Review Board (HIRB, No. 2023‐938)

## Consent

All patients who participated in this study signed informed consent form approved by the Huashan Institutional Review Board (HIRB).

## Conflicts of Interest

The authors declare no conflicts of interest.

## Data Availability

The data that support the findings of this study are available from the corresponding author upon reasonable request.
